# The regulation of tumor suppressor protein, p53, and estrogen receptor (ERα) by resveratrol in breast cancer cells

**DOI:** 10.18632/genesandcancer.125

**Published:** 2016-11

**Authors:** Julieta Saluzzo, Kelly M. Hallman, Katie Aleck, Brigitte Dwyer, Meghan Quigley, Viktoria Mladenovik, Amy E. Siebert, Sumi Dinda

**Affiliations:** ^1^ School of Health Sciences, Oakland University, Rochester, MI, USA; ^2^ Prevention Research Center, Oakland University, Rochester, MI, USA

**Keywords:** Breast cancer, tumor suppressors, p53, estrogen, antiestrogens

## Abstract

Resveratrol (RES) is a natural antioxidant found abundantly in grapes, peanuts, and berries, and is known to possess anti-tumorigenic properties. However, there is a noticeable lack of studies on the mechanistic effects of Resveratrol on tumor suppressors. Previous studies from our laboratory have shown the tumor suppressor protein p53 and estrogen receptor-alpha (ERα) to be possible molecular targets for RES. In this study, the anti-estrogenic effects of RES were analyzed on the expression of ERα and p53. The breast cancer cells grown in stripped serum were treated with 60 μM RES, as the optimum concentration based on data obtained from a concentration study using 1-100 μM RES. Our studies indicate that RES caused a decrease in the levels of protein expression of p53 and ERα as compared to the control. Increasing concentrations of RES caused a four-fold decrease in cell number in comparison to estradiol. RES, in conjunction with ICI 182,780 (ICI), caused a down-regulation of both p53 and ERα as compared to the control. These observed effects on cell proliferation and regulation of both p53 and ERα by RES may lead to further understanding of the relationship between tumor suppressor proteins and steroid receptors in breast cancer cells.

## INTRODUCTION

Breast cancer is the second leading cause of death for women in the United States [1]. According to the American Cancer Society, there are an estimated 60,290 new cases of female breast carcinoma in situ to be diagnosed in 2015. This accounts for about 20% of all breast cancer in women, and DCIS will account for the vast majority (83%)[2]. The definition of DCIS, or ductal carcinoma in situ, is the pre-malignant proliferation of neoplastic epithelial cells contained within the lumen of mammary ducts [3]. If left untreated, DCIS could eventually evolve into invasive breast cancer, thus recognized as a true cancer precursor [2]. Invasive breast cancer (IBC) is the final stage of a long transformation of increasingly abnormal premalignant stages of DCIS [3]. In 2011, there were an expected 60,000 new cases of DCIS, and about a third were expected to evolve into IBC [4]. These numbers could be brought down significantly by improving the ability to detect, diagnose, and treat DCIS [4]. A potential treatment option is hormone therapy, which is most effective in breast cancers that possess estrogen receptors (ER) [2]. Numbers indicate that 50-75% of DCIS lesions express ER, which correlate with DCIS grade [1]. Progress will be based on a detailed understanding of molecular mechanisms responsible for the development and progression of DCIS to IBS and on a greater understanding of ER, its various mechanisms of action, its activation at the genetic level, and what these findings may mean as prognostic implications [1].

Resveratrol (3,5,4′-trihydroxy-*trans*-stilbene) is a phytoalexin commonly found in red wine and in foods including peanuts, blueberries, cranberries, and grapes [5]. This compound has been shown to possess antioxidant and anti-inflammatory properties [6, 7], as well as an ability to inhibit the initiation, promotion, and progression of carcinogenesis [8, 9]. In contrast, at concentrations comparable to those required for its reported anti-carcinogenic effects, this phytoestrogen has also been reported to act as a super-agonist for the estrogen receptor (ER) in ER-positive breast cancer cells [8, 10]. However, several studies suggest that low concentrations (1 - 20 μM) of RES exert an ER-mediated mitogenic effect in ER-positive breast cancer cells, but that high concentrations (60-100 μM) of RES exhibit ER-independent anti-proliferative activity [10, 11]. Studies using MCF-7 breast cancer cells show that with lower concentrations (10^−7^ to 10^−5^ mol/l) of RES, there is a reduction of cellular proliferation in a dose and time-dependent manner, via an ER-dependent mechanism [12, 13]. Yet, studies show that the phytoestrogen is also capable of maintaining normal breast cell survival via ER-independent or other mechanisms [13]. Resveratrol was also shown to increase apoptotic cells in both T-47D and MCF-7 breast cancer cells [13-16]. There is also evidence from studies that show p53 induction in MCF-7 and T-47D breast cancer cells is associated with the anti-proliferative effects of Resveratrol. Yet, Resveratrol-induced cytotoxicity in MCF-7 breast cancer cells was also partially mediated by p53[17]. Some studies suggest that Resveratrol not only controls activation of p53, but that it also increases protein levels of p53[14-17]. Previous studies from our laboratory have shown that the addition of exogenous 17β-estradiol (E_2_) causes an increase in cellular proliferation of T-47D breast cancer cells, as well as accumulation of the tumor suppressor protein p53[18, 19]. In other studies, when 17β-estradiol (E_2_) was introduced, the expression of ERα was significantly increased, and E_2_ induced cell proliferation [12, 16, 20].

The focus of this present study is to investigate the effects of Resveratrol in combination with ERα agonist (E_2_), endocrine disrupter (BPA), and anti-estrogens (ICI and Tamoxifen) on the expression of p53 and ERα in T-47D breast cancer cells.

## RESULTS

### Regulation of ERα and p53 levels by Resveratrol and BPA, E2, and other anti-estrogens

In order to observe the effects of exogenous steroids, trace amounts of endogenous steroids, including E_2_, can effectively be removed from culturing media by treatment of the serum with charcoal suspension, a process known as “stripping” of the media. Previous experiments conducted in our laboratory have determined that upon treatment with E_2_, the levels of ERα and p53 in T-47D cells cultured in stripped serum are maximal at 24 hours. In this particular study, we compared the effects of Resveratrol (RES) in the presence of E_2_, BPA, ICI, and Tamoxifen (TAM) on the levels of ERα and p53. Equal amounts of cells were plated in whole serum (containing endogenous E_2_) for two days, and for six days thereafter maintained in media containing stripped serum. The cells were treated with hormones and anti-hormones for 24 hours.

**Figure 1:**
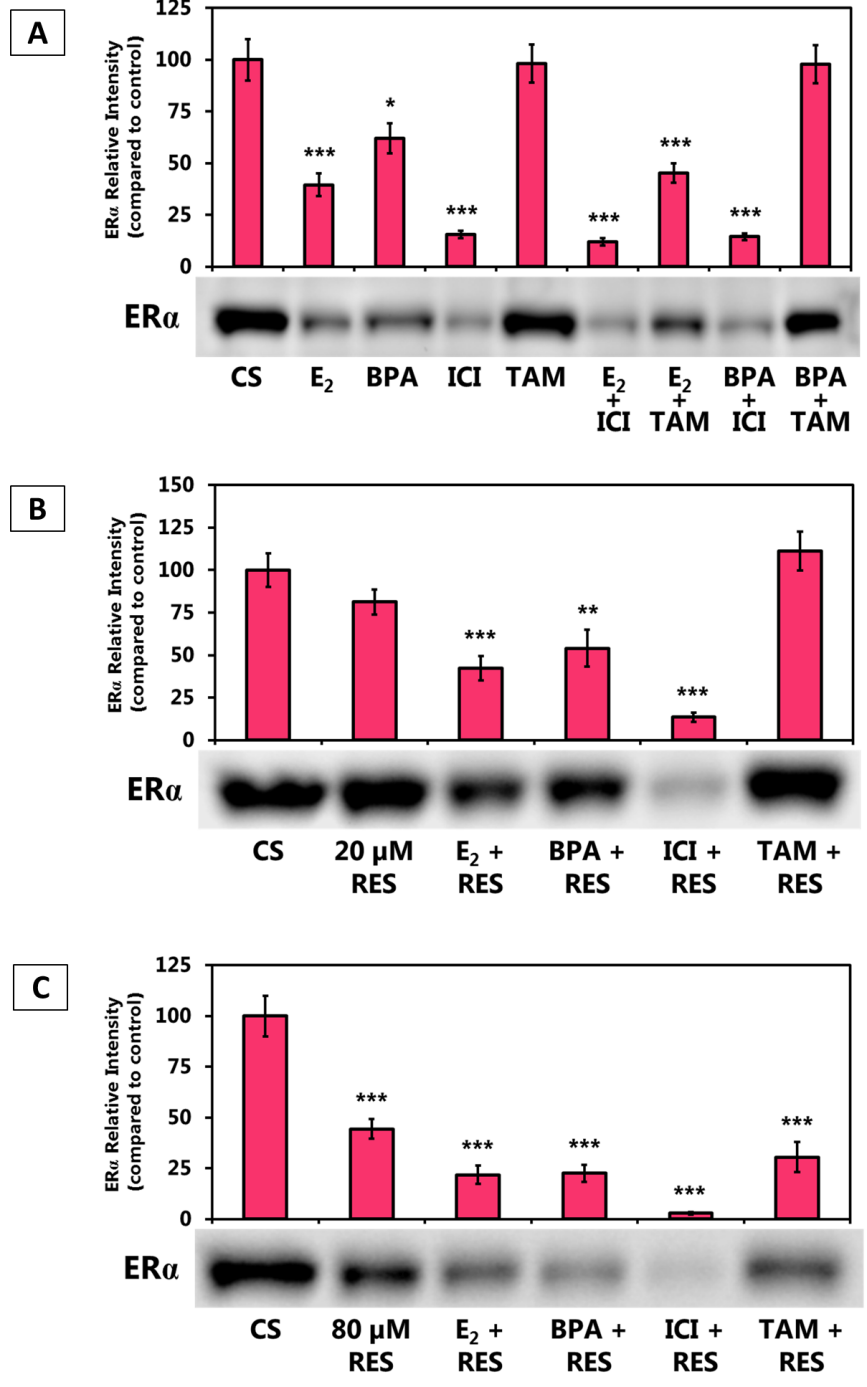
**(A-C).** Effects of Estrogen Receptor Agonists and Antagonists in Combination with Resveratrol on the Levels of ERα Protein Expression. T-47D cells were treated with 20 and 80 μM RES, 10 nM E2, 600 nM BPA, 1 μM ICI and 1 μM tamoxifen (TAM) either alone or in combination for 24 hours. Western blot analyses of ERα. Protein extraction was done with a RIPA buffer and quantification was performed using the BioRad Bradford Assay kit. The supernatant was denatured (3 minutes, 85°C) and 30 μg aliquots of total protein/lane were loaded on a 7.5% polyacrylamide gel under denaturing conditions and electrophoresed for the separation of proteins. The relative intensities of each band were compared to the control. Representative blots from three independent experiments are shown.

Figure 1A shows the effects of the estrogens and anti-estrogens, alone and in combination, on ERα protein levels. Effects on ERα protein levels were similar when estrogens, anti-estrogens, and BPA were used alone or when used in latter combination. ERα protein levels were up-regulated when an estrogen/anti-estrogen combination was used with a low concentration of RES (20 μM), except for ICI (Figure 1B). ERα protein levels were down-regulated when an estrogen/anti-estrogen combination was used with anti-proliferative (80 μM) RES concentrations (Figure 1C).

**Figure 2:**
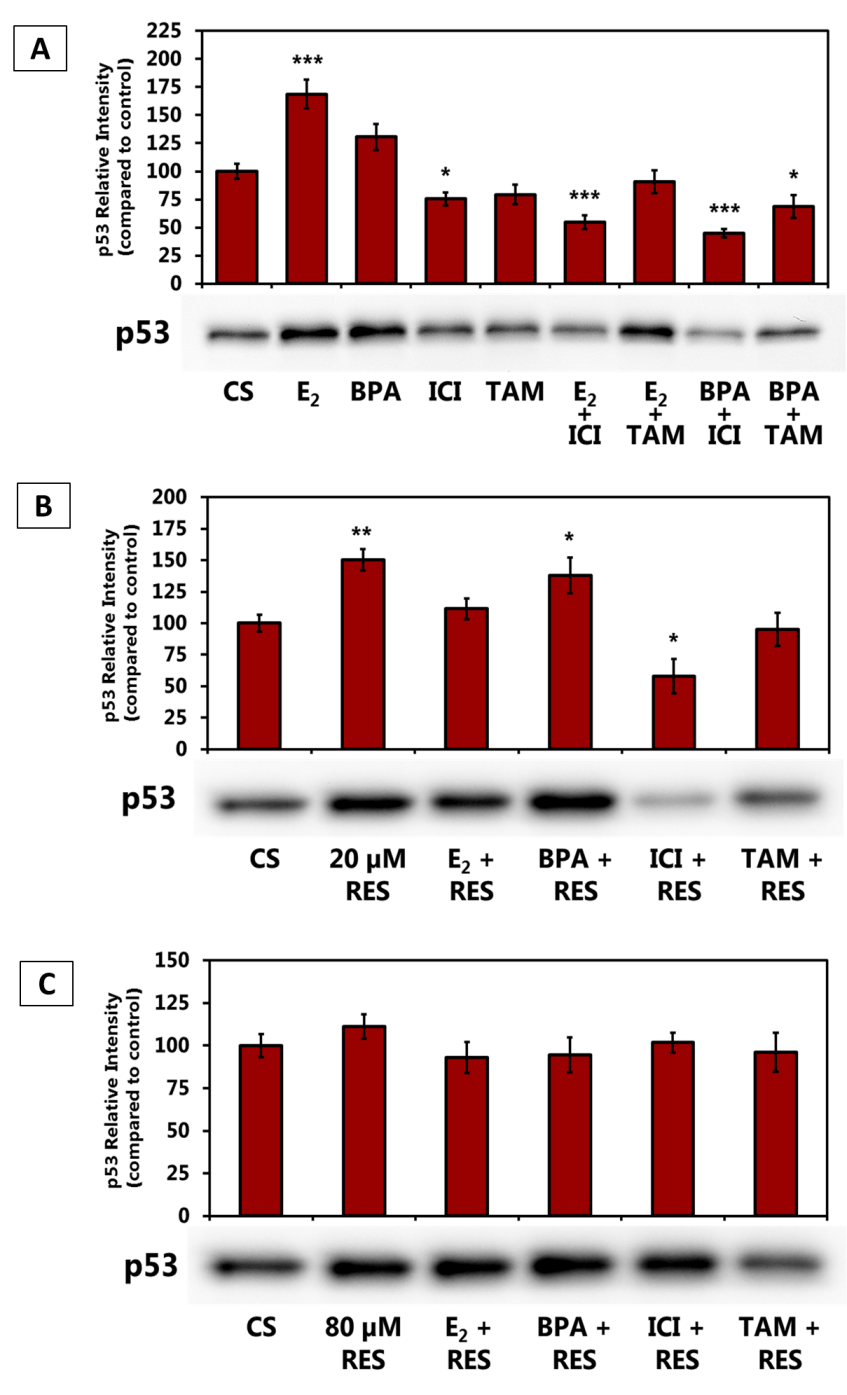
**(A-C).** Effects of Estrogen Receptor Agonists and Antagonists in Combination with Resveratrol on the Levels of p53 Protein Expression. T-47D cells were treated with 20 and 80 μM RES, 10 nM E2, 600 nM BPA, 1 μM ICI and 1 μM tamoxifen (TAM) either alone or in combination for 24 hours. Western blot analyses of p53. Protein extraction was done with a RIPA buffer and quantification was performed using the BioRad Bradford Assay kit. The supernatant was denatured (3 minutes, 85°C) and 30 μg aliquots of total protein/lane were loaded on a 7.5% polyacrylamide gel under denaturing conditions and electrophoresed for the separation of proteins. The relative intensities of each band were compared to the control. Representative blots from three independent experiments are shown.

Figure 2A shows the effects of estrogen, anti-estrogens, and BPA, alone and in combination, on p53 protein levels of expression. Addition of exogenous E_2_ increased the expression level of p53, yet upon addition of ICI, the effect of the estrogen was blocked. ICI also blocked the effect of BPA on the level of expression of p53. TAM was not as effective when used in combination with E2 or BPA. When 20 μM RES was used with the ligands, the level of expression of the p53 protein was inhibited by ICI (Figure 2B). The p53 protein levels were not altered when anti-proliferative (80 μM) concentrations were used with estrogen/anti-estrogen combination (Figure 2C).

### Effects of thyroid hormone on the ERα and p53 levels after treatment with charcoal treated FBS

**Figure 3:**
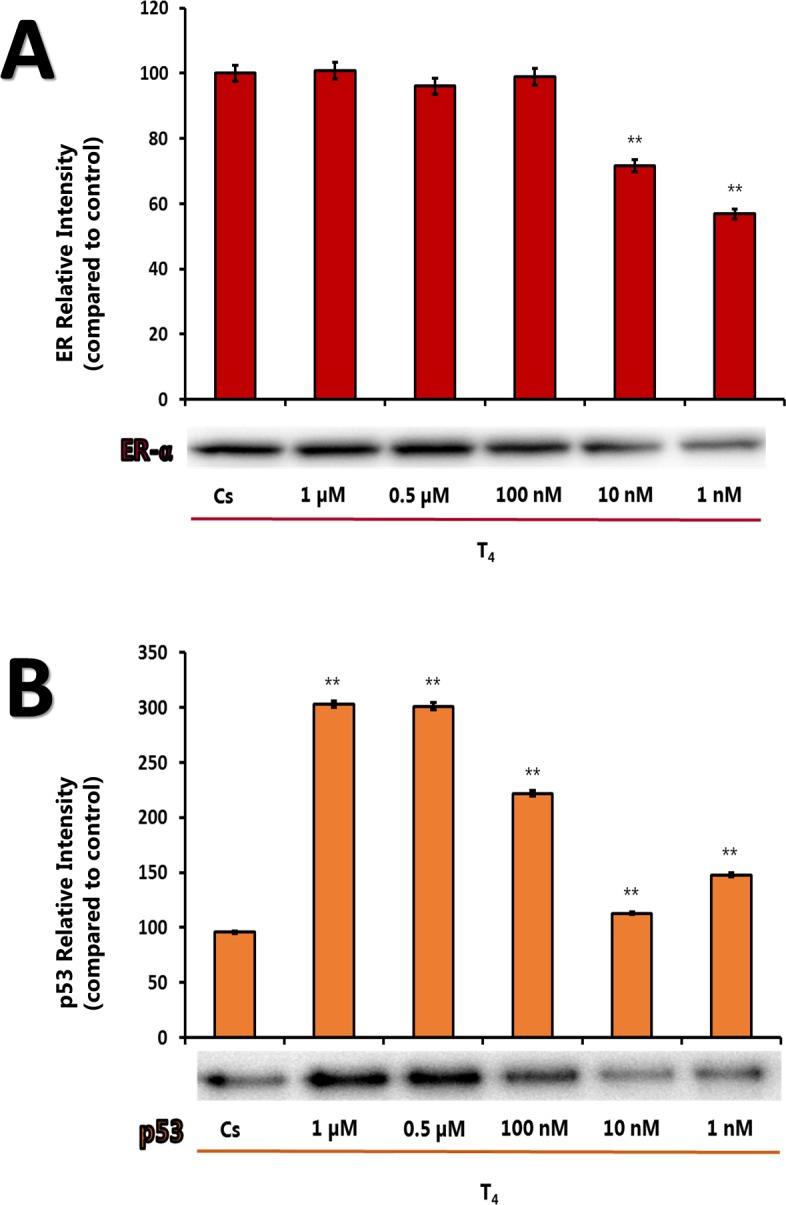
**(A-B).** Effects of Thyroid Hormone on ERα and p53 Levels. T-47D cells were treated with varying concentrations (1 μM-1 nM) of T4 following treatment with charcoal stripped FBS for 24 hours. Western blot analyses of ERα and p53 were performed. Protein extraction was done with a RIPA buffer and quantification was performed using the BioRad Bradford Assay kit. The supernatant was denatured (3 minutes, 85°C) and 30 μg aliquots of total protein/lane were loaded on a 7.5% polyacrylamide gel under denaturing conditions and electrophoresed for the separation of proteins. The relative intensities of each band were compared to the control.

Figures 3A and 3B demonstrate the effects of different concentrations of thyroid hormone (T_4_) on the levels of p53 and ERα. Cells were treated with different thyroid hormone (T_4_) in the presence of stripped serum as described previously. T_4_ at higher concentrations (0.5 – 1.0 μM) caused an E_2_-like increase in p53 levels when compared to its effects on cells grown in the stripped medium. At similar high concentrations of T_4_ (0.5 – 1.0 μM), the level of ERα appeared to increase similar to the control (stripped). At lower concentrations (1 to 10 nM), T_4_ did not have significant effect on the levels of p53 and ERα.

### Regulation of ERα, Integrin, Lysine p53 (activated form) and p53 levels by Resveratrol

**Figure 4:**
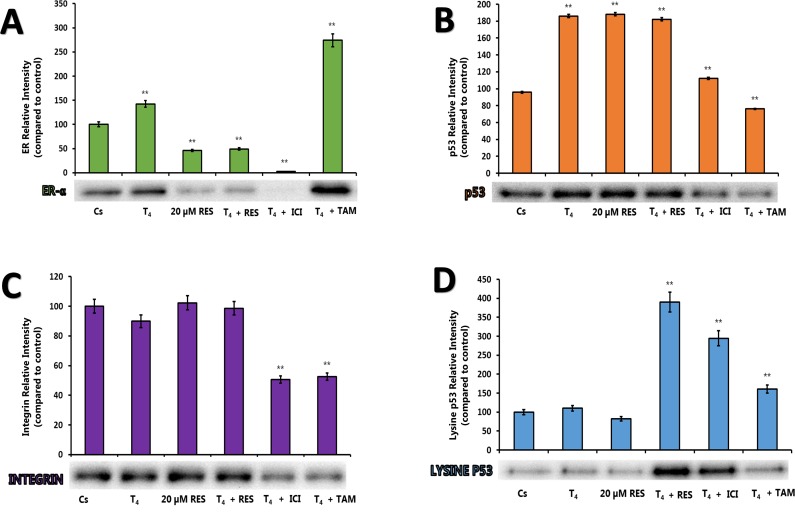
**(A-D).** Effects of ERα, Integrin, Lysine p53 (activated form) and p53 Levels by 20 μM Resveratrol. T-47D cells were treated with 20 μM RES, 1 μM T4, 1 μM ICI and 1 μM tamoxifen (TAM) either alone or in combination for 24 hours. Western blot analyses of ERα, p53, Integrin, and Lysine p53 were performed. Protein extraction was done with a RIPA buffer and quantification was performed using the BioRad Bradford Assay kit. The supernatant was denatured (3 minutes, 85°C) and 30 μg aliquots of total protein/lane were loaded on a 7.5% polyacrylamide gel under denaturing conditions and electrophoresed for the separation of proteins. The relative intensities of each band were compared to the control.

Figures 4A-4D represent the effects of low concentration of RES (20 μM) with and without T_4_, and anti-estrogens in the presence of stripped serum as described previously. Treatment with T_4_ caused an up-regulation of ERα and p53 expression, while displaying no effect in integrin and lysine p53 expression compared to control. When the cells were treated with 20 μM of RES, there was down-regulation of ERα and lysine p53 expression. Similar treatment caused an up-regulation of p53 and integrin. These effects were inhibited when they were treated in the presence of anti-estrogens.

**Figure 5:**
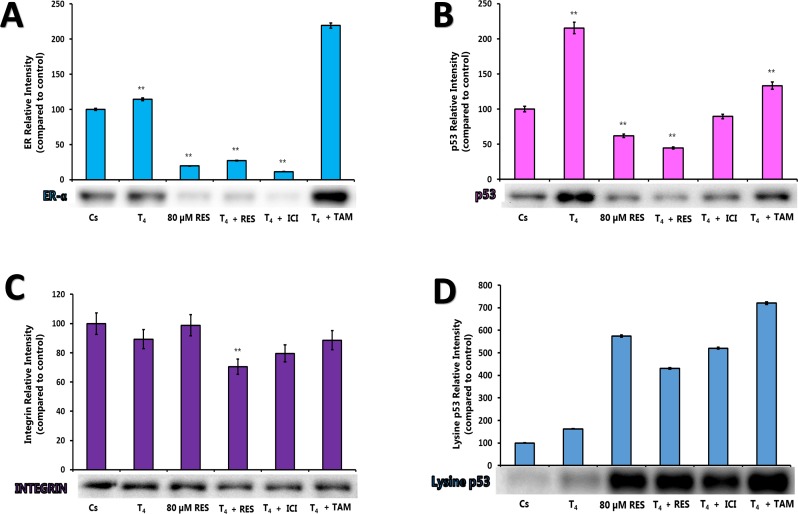
**(A-D).** Effects of ERα, Integrin, Lysine p53 (activated form) and p53 Levels by 80 μM Resveratrol. T-47D cells were treated with 80 μM RES, 1 μM T_4_, 1 μM ICI and 1 μM tamoxifen (TAM) either alone or in combination for 24 hours. Western blot analyses of ERα, p53, Integrin, and Lysine p53 were performed. Protein extraction was done with a RIPA buffer and quantification was performed using the BioRad Bradford Assay kit. The supernatant was denatured (3 minutes, 85°C) and 30 μg aliquots of total protein/lane were loaded on a 7.5% polyacrylamide gel under denaturing conditions and electrophoresed for the separation of proteins. The relative intensities of each band were compared to the control.

Figures 5A-5D represent the effects of RES (80 μM) with and without T_4_ and anti-estrogens in the presence of stripped serum as described previously. Treatment with T_4_ caused an up-regulation of ERα, integrin, and p53 expression, while displaying down-regulation in lysine p53 expression. When the cells were treated with 80 μM of RES, there was down-regulation of ERα, and p53 expression. Similar treatment caused an up-regulation of integrin and lysine p53. These effects are opposite of the treatment in higher concentration. The expression of integrin was slightly blocked in the presence of anti-estrogens, whereas the lysine p53 expression was up-regulated three folds in presence of anti-estrogens.

### Regulation of ERα and p53 levels by Resveratrol: Concentration Dependency

**Figure 6:**
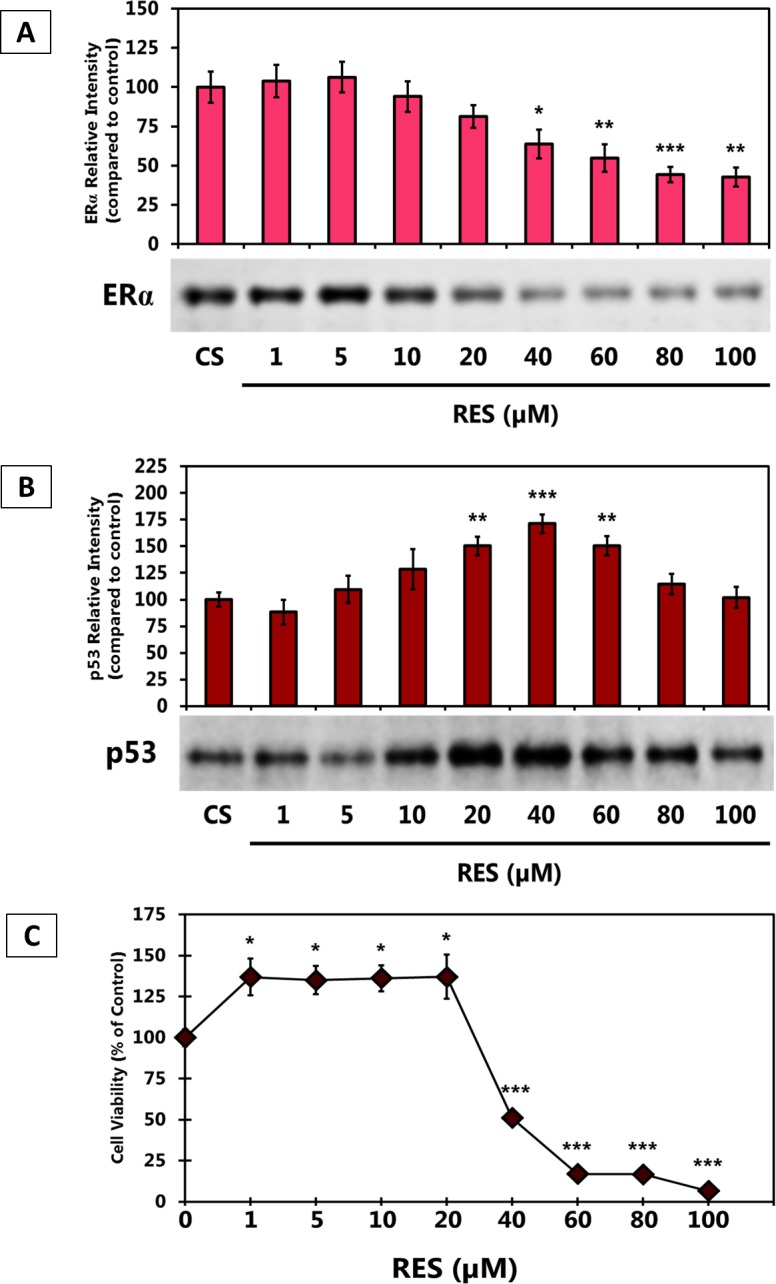
**(A-C).** Effects of Resveratrol on T-47D Cell Viability and Levels of p53 and ERα Protein Expression. (A and B) T-47D cells were treated for 24 hours with 1 - 100 μM RES and subjected to SDS-PAGE and Western blot analysis. The relative intensities of each band were compared to the control. Representative blots from three independent experiments are shown. (C) T-47D cells were treated for 6 days with 1 - 100 μM Resveratrol (RES) and cell viability was determined by propidium iodide staining and image cytometry.

Figure 6A demonstrates the effects of different concentrations of RES on the levels of ERα. At lower concentrations (1 - 20 μM), RES did not affect the ERα expression levels as compared to the control. At higher concentrations of RES (60 - 100 μM), the ERα protein level of expression declined. Figure 6B demonstrates the effects of different concentrations of RES on the levels of p53. RES (1 - 40 μM) appears to increase the level of p53. Higher concentrations of RES (60 - 100 μM) less effectively regulate the level of p53.

### Effects of Resveratrol on the cell viability of T-47D Cells

Figure 6C demonstrates the effects of different concentrations of Resveratrol on cell viability. 1 - 20 μM RES increased breast cancer cell viability while 40 - 100 μM RES decreased cell viability (anti-proliferative). Our laboratory subsequently assessed the effects of RES, E_2_, BPA, and the anti-estrogens, ICI and TAM, on the T-47D cell viability in order to correlate this data with the cellular levels of ERα and p53, respectively.

**Figure 7:**
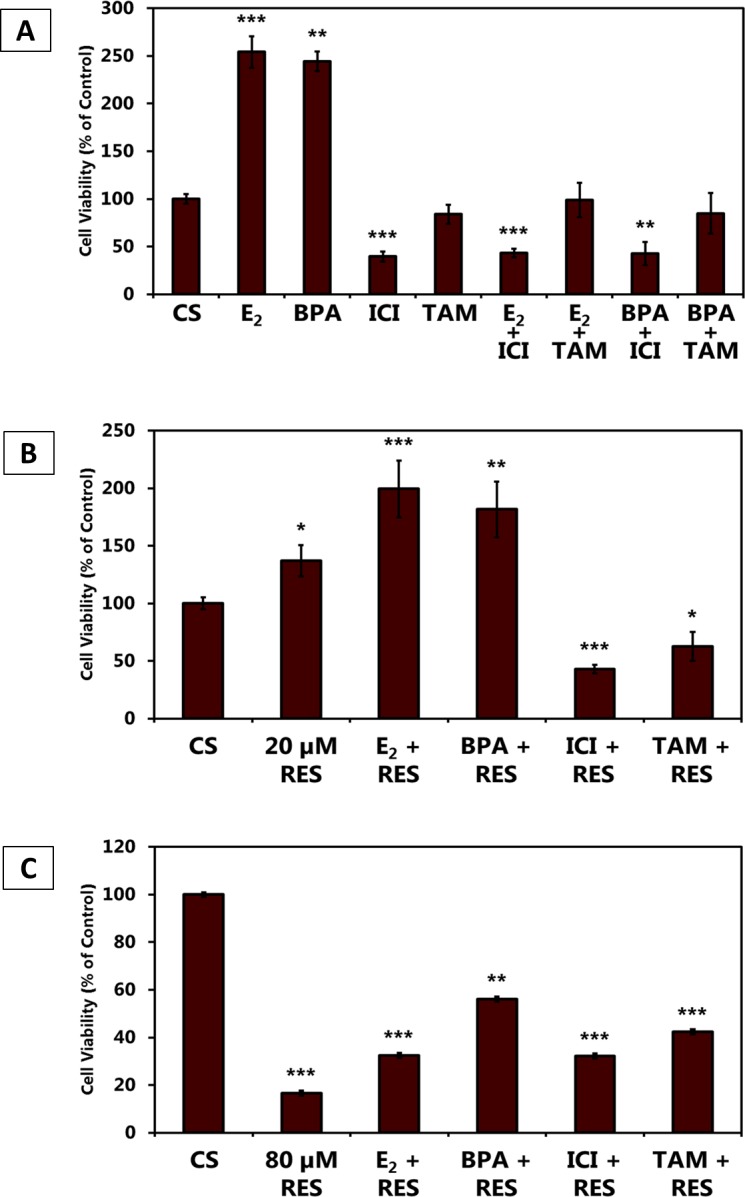
**(A-C).** Effects of Estrogen Receptor Agonists and Antagonists in Combination with Resveratrol on T-47D Cell Viability. T-47D cells were treated with 20 and 80 μM RES, 10 nM E2, 600 nM BPA, 1 μM ICI and 1 μM tamoxifen (TAM) either alone or in combination for 6 days. Cell viability was determined by propidium iodide staining and image cytometry.

Figure 7A demonstrates the effects of E_2_, BPA, and the anti-estrogens (ICI & TAM) on cell viability, alone and in combination. ICI blocked the effects of E_2_ and BPA, while TAM did not appear to affect cell viability as compared to the control. The mitogenic effects of 20 μM RES appear to be sensitive to anti-estrogens (Figure 7B). RES (80 μM) seems to reverse the effects of Estrogen and BPA, while eliciting no effect when combined with anti-estrogens (Figure 7C).

### Confocal Microscopy (Figure. 8)

**Figure 8:**
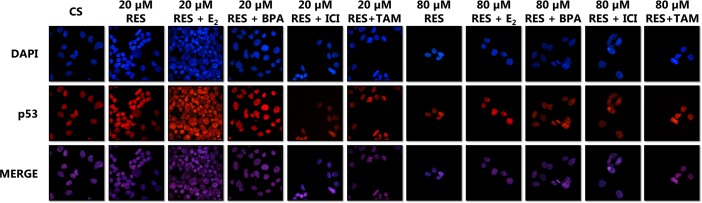
Effects of Resveratrol on the Cellular Localization of p53 Images were acquired as described in Materials and Methods.

To determine if the effects of RES on the level of p53 correlated the expression with alterations in the cellular localization of the tumor suppressor proteins, immunolabeling of p53 protein in T-47D cells was performed, followed by laser-scanning confocal microscopy. Consistent with the transcriptional function of this nuclear phosphoprotein, results in Figure 8 reveal that p53 is cytolocalized in the nuclei of T-47D cells cultured in 5% DCC-FBS. This nuclear localization appears predominantly dispersed throughout the nuclear compartment, which can be seen in the DAPI (nuclear counterstain) and p53 merged images. Treatment with E_2_ and RES increased the intensity of the nuclear staining of p53 as detected by immunofluorescence. When the cells were exposed to RES, the degree of immunofluorescence was greater than observed in the control (C).

### RT-qPCR (Figure. 9)

**Figure 9:**
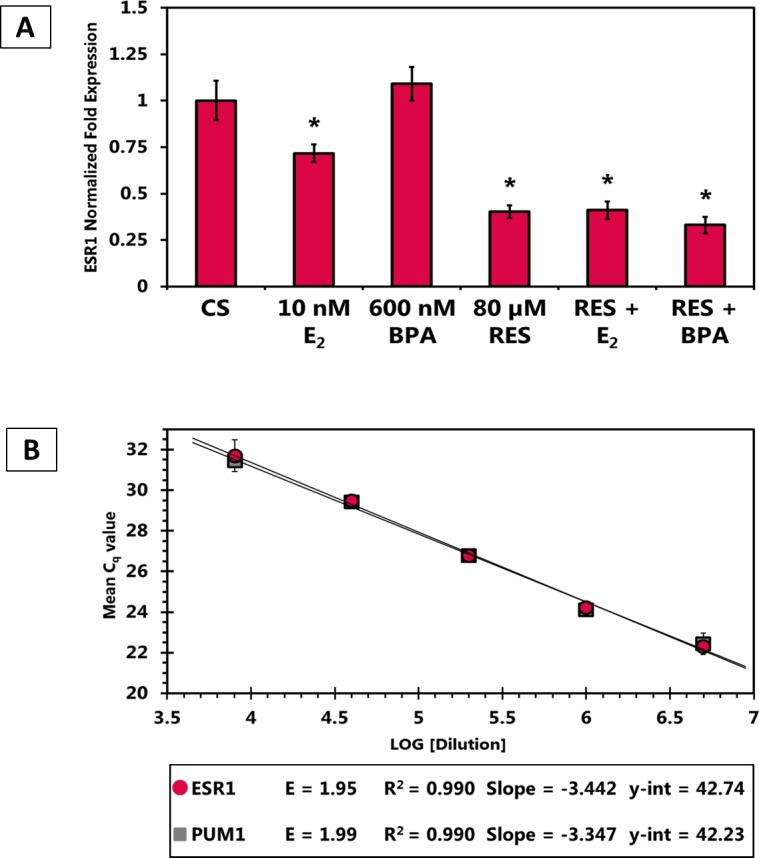
Effect of RES, E2 and BPA alone and in combination on ESR1 levels in T-47D breast cancer cells (A) ESR1 mRNA levels determined by reverse transcription quantitative real time PCR (RT-qPCR). T-47D cells were treated in the presence or absence of 80 μM Res, 10 nM E_2_ and/or 600 nM BPA for 24 hours. Results are shown as the mean ± SEM of at least three independent experiments with three replicates in each experiment. The asterisk above the bars, represent significant difference when compared to the control at p< 0.05 (Kruskal-Wallis Test followed by post-hoc analysis using Mann-Whitney U-Test). (B) Calculation of PCR efficiencies. Real-time PCR efficiencies of reference (PUM1) and target gene (ESR1) were determined. Cq was plotted against the log amount of cDNA input. Amplification efficiencies were calculated according to the equation E = 10(−1/slope). ESR1, Estrogen Receptor α; PUM1, Pumilio homolog 1.

To study the effects of RES on the levels of ERα under various conditions, RT-qPCR was utilized to detect the mRNA expression levels of ESR1. Biological replicates from three independent experimental reproductions were separated and amplified in triplicate for the GOI and reference genes. In Figure 9A, the Y-axis represents the mRNA fold expression, normalized to the expression of the reference gene PUM-1, whereas the X-axis represents the treatment conditions for 24 hours. The ESR1 mRNA levels were significantly reduced with 10 nM E2, 80 μM RES, and RES in combination with E2 and BPA. The effects observed by RES alone or RES combination with Estrogen or BPA induced a 50% decrease in ESR1 expression levels as compared to the DMSO-treated control. Figure 9B represents the real-time PCR efficiencies of target gene (ESR1) and reference gene (PUM1) that were determined. Cq was plotted against the log amount of cDNA input and the relationship between Cq values and RNA concentration was calculated by linear regression to find a slope and intercept that predicts cDNA amounts and correlation coefficient (R2). Amplification efficiencies (E) were calculated according to the equation E = (10(−1/slope) − 1) × 100 and are expressed as a percentage.

## DISCUSSION

Even though studies have reported that stripping fetal bovine serum removed important factors like thyroid hormones and vitamins which may affect the growing conditions of breast cancer cells [21], our studies demonstrate that addition of T_4_ (L-thyroxine) with concentrations of 0.001 to 1 μM after stripping did not significantly alter the levels of ERα or p53. However, addition of T_4_ showed increased levels of p53 and ERα expression which is similar to the estrogen dependent expression [22]. Davis et al. have shown that Resveratrol has a binding site to a receptor site on the integrin αvΔ3 and the binding of Resveratrol may induce apoptosis in breast cancer cells [23]. We have observed similar results as Davis et al.; RES (20 and 80 μM) induced integrin expression, which may explain p53-dependent apoptotic mechanisms in the T-47D breast cancer cell line. Combination of T_4_ with the higher concentration of RES decreased expression levels of integrin compared to RES alone. These effects were inhibited with the treatment of anti-estrogens. Acetylation of p53 is mediated by p300 and CBP acetyltransferases. The accumulation of p53 in a stress response is mainly due to acetylation. Human p53 becomes acetylated at Lys382 (Lys379 in mouse) in vivo to enhance p53-DNA binding. The histone acetyltransferases p300 and PCAF can acetylate p53 in vitro at Lys382 and Lys320, respectively [24]. Lys382 becomes acetylated in vivo following DNA damage to allow enhanced p53-DNA binding [25]. This activation also influences the apoptosis mechanism driven by p53 accumulation. We have observed similar results with 20 and 80 μM with RES compared to the control, where the levels of lysine p53 were down-regulated which may suggest another hormone-independent mechanism. Cells treated with a combination of T_4_ and anti-estrogens up-regulated the levels of lysine p53, which may suggest an apoptotic mechanism. Treatment with 1 - 20 μM RES significantly increased T-47D cell viability with a concomitant increase in p53 protein levels. However, higher concentrations of RES (40 - 100 μM) significantly decreased the cell number and became less effective in its ability to regulate p53 protein levels. Other studies have shown that RES, at increasing concentrations ranging from 10 - 100 μM, induced a dose-dependent inhibition of cell growth, with a statistically significant reduction of T-47D cells, and a post-transcriptional p53 dose-dependent induction [26]. In contrast, other studies did not present any significant changes in viability with lower concentrations of RES (0.1 μM, 1 μM, and 10 μM), as compared to the control group. Yet, apoptotic cells increased with 0.1μM RES [20]. When cells were exposed to 25 μM and 50 μM concentrations of RES, there was inhibition of growth by >55% and >60%, respectively. This resulted in a p53 induction of a dose-dependent manner. Therefore, the growth inhibition induced by RES in breast cancer cells is linked to induction of apoptosis [15]. Our own findings challenge these observations, demonstrating cell proliferation when cells are treated with low RES concentrations (1 - 20 μM), and a less effective regulation of the p53 protein when treating cells with higher concentrations of RES (40 - 100 μM).

The level of ERα protein expression was significantly down-regulated with 40 - 100 μM RES, with no effect observed at lower concentrations, which induced mitogenic effects in T-47D cells. Previous studies have shown that the expression level of ERα was significantly up-regulated in cells treated with E_2_ and a combination of E_2_ and RES at low concentrations, but not with RES alone [20]. At a lower concentration (20 μM), mitogenic effects of RES are sensitive to BPA and ICI, whereas at a higher anti-proliferative concentration (80 μM) RES reversed the effects of E_2_ and BPA, while enhancing the effect of ICI. Data obtained through our studies show that higher concentrations of RES inhibit the stimulatory effect of E_2_ on cell growth, inducing similar effects to that of RES alone [16]. In accordance to our findings, which showed an enhanced down-regulation of ERα expression when 80 μM RES and ICI were used in combination, another study found that when adding ICI, the phytoestrogen/anti-estrogen combination increased apoptotic cells and reduced the number of cell colonies, with RES enhancing the effect of ICI. This effect is exerted via an ER-independent pathway. RES may also act in part via an ER-dependent mechanism due to its stimulation of endogenous ER transcription [16]. Interestingly, our findings demonstrated that the effect of 20 μM RES on p53 protein level was inhibited by ICI. Effects on the level of ERα protein induced by these estrogen and anti-estrogen combinations reflected effects similar to those observed when the estrogens and anti-estrogens were used alone. In other studies, when cells were treated with a low concentration of RES and E_2_, the ERα expression was significantly increased. Our data supported these study findings by showing a statistically significant increase when a combination of 20 μM of RES and E_2_ were used. However, the opposite was observed when the cells were treated with RES alone, suggesting an ER-dependent mechanism for growth inhibition [12, 13]. As previously mentioned, our findings showed no significant up-regulation of the ERα protein when low concentrations of RES (1 - 20 μM) were used to treat the cells. In contrast, the combination of estrogens and anti-estrogens with an anti-proliferative concentration of RES down-regulated ERα protein levels, but was ineffective in altering p53 protein levels.

Previous studies have shown that at high concentrations of RES (100 and 200 μM), cells exhibited a dose-dependent increase in the total level of p53 protein level. Therefore, it can be inferred that p53 partially mediated RES-induced apoptosis in breast cancer cells [17]. Other studies have concluded that RES controls p53 activity assessed by an increase in phosphorylation [14, 26]. Tumor suppressor factor p53 is also a transcription factor, strongly enhancing the rate of transcription of other important genes that play an important role in cell cycle arrest and apoptosis, such as p21. One study concluded that RES demonstrated a dose-dependent induction of both p53 and p21, seen at both the mRNA and protein levels. This indicates that the cell cycle could be affected due to alterations of such important genes [15]. As previously mentioned, our studies did not exhibit an effective regulation of the p53 protein when cells were treated with higher concentrations of RES, eliciting a biphasic effect of different concentrations of RES and its effect on the level of expression of p53.

ESR1 mRNA levels were significantly reduced with 10 nM E2, 80 μM RES, and RES in combination with E_2_ and BPA. Consistent with our findings, RES seems to inhibit proteins that are involved in translation [26]. Based on the observations of biphasic effects on cell viability and p53 protein expression, along with concentration-dependent alterations in the sensitivity of both estrogens and anti-estrogens in our results, and with the results from the literature, there is a possibility that a dual mechanism for RES actions is both ER-dependent and independent.

In conclusion, Resveratrol may be a modulator for a receptor that exhibits concentration-dependent functional selectivity as well as cross-talk with ERα. Regardless, it is clear that Resveratrol regulates p53 and ERα on a molecular level. Whether this regulation is mediated by ERα or by an alternative inhibitory cross-talk mechanism calls for future investigations. Understanding the dose-response relationship of Resveratrol may aid in the development of more selective estrogen receptor agonists and antagonists, as well as potentially blocking the effects of endocrine disrupting chemicals, such as BPA. Further studies are warranted as our results support the potential for Resveratrol as a preventative measure against breast cancer initiation and progression.

## METHODS AND MATERIALS

### Cell Culture and Treatment with Ligands

T-47D human breast cancer cells (ATCC® HTB-133TM) were routinely cultured in RPMI-1640 (Hyclone, Logan, UT), containing 2 mM L-glutamine, 25 mM Hepes, antibiotic antimycotic solution (100 units/ml penicillin, 0.1 mg/ml streptomycin, and 0.25 mg/ml amphotericin B) (Hyclone), and 0.14 IU/ml insulin (Sigma) supplemented with 10% (v/v) fetal bovine serum (FBS) (Hyclone) and incubated at 37°C in the presence of 5% CO2. In all studies, T-47D cells were sub-cultivated into RPMI-1640 media containing the above components with alterations in the type and percentage of FBS utilized. Cells were plated in 10% FBS media and allowed to attach and grow for 48 hours. To ensure steroid-free treatment conditions, the cell culture medium was changed to contain 5% FBS stripped with Dextran-coated charcoal (DCC-FBS) as described [27], which depletes the serum of small heterocyclic molecules including serum growth factors. Prior to treatment with ligands, the cells were cultured in 5% DCC-FBS for a total of 4 days, with fresh medium added every 48 hours. On the fourth day, semi-confluent cells were treated with various ligands and/or inhibitors for the time periods indicated in the figure legends.

### SDS-PAGE and Western Analyses

Protein extraction was done with a RIPA buffer and quantification was performed using the BioRad Bradford Assay kit [19]. The supernatant was denatured (3 minutes, 85°C) and 30 μg aliquots of total protein/lane were loaded on a 7.5% polyacrylamide gel under denaturing conditions and electrophoresed for the separation of proteins. Proteins were wet transferred to Immobilon-P polyvinylidene fluoride (PVDF) membrane (Millipore, Bedford, MA) by using a Bio-Rad trans blot cell (100 V, 1 hour) in a tris-glycine buffer system containing 0.025% SDS and 15% methanol. Membranes were blocked for 1 hour in 5% (w/v) non-fat dry milk (NFDM) in TBS-T (20 mM Tris-HCl, 140 mM NaCl, pH 7.4, 0.1% (v/v) Tween 20) and incubated with primary antibody anti-ERα clone F-10 (Santa Cruz), anti-p53 (Santa Cruz), anti-integrin αvΔ3 (Cell Signaling) & anti-lysine p53 (Cell Signaling) diluted 1:500 in 5% (w/v) NFDM in TBS-T for 2 hours. After washing with TBS-T (3 × 10 minutes) and incubation with horse-radish peroxidase (HRP)-conjugated goat anti-mouse IgG2a secondary antibody (Santa Cruz) diluted 1:1000 in 5% (w/v) NFDM in TBS-T for 1 hour, blots were developed using Amersham ECL Prime (GE Healthcare Biosciences, Piscataway, NJ), and imaged using the Bio-Rad ChemiDoc XRS+ system. After immunoblotting, the PVDF membranes were then stained with Coomassie blue to ensure the correct normalization against total protein levels and full transfer of protein. The Western blots were subjected to quantification of the protein band density using the Image Studio Lite program version 3.1 (LI-COR Biosciences, Lincoln, NE).

### RNA Extraction and cDNA Synthesis

Total RNA was extracted from T-47D cells using ‘Trizol’ reagent (Invitrogen, Life Technologies, USA) according to manufacturer's protocol. Total RNA was re-suspended in 20 μl RNase free water and the RNA concentration and purity was determined by Eppendorf Biophotometer. RNA integrity was confirmed by gel electrophoresis using 1% agarose with ethidium bromide. To eliminate genomic DNA, 1 μg of total RNA from all samples were further treated with DNase I (Invitrogen) according to manufacturer's protocol. gDNA-free total RNA (50 ng) was reverse transcribed using iScript cDNA Synthesis Kit (Bio-Rad Laboratories, Hercules, CA) according to manufacturer's protocol. Reverse transcription was performed for 5 minutes at 25°C, 30 minutes at 42°C, and 85°C for 5 minutes. RT samples were used immediately or stored at −20°C until further use.

### Quantitative Real-Time Analysis

Quantitative real-time PCR of reverse transcribed cDNA (RT-qPCR) was performed in 384-well format in the Bio-Rad CFX384 Real Time System. The concentration of cDNA refers to, and is derived from, the concentration of RNA that was originally synthesized in the cDNA synthesis reaction described above. The real-time PCR mixtures consisted of 1.0 μL cDNA template (corresponding to 1 ng cDNA/well), 400 nM specific sense primer, 400 nM specific antisense primer, RNase/DNase-free water, and 1x SsoAdvanced SYBR Green Supermix (Bio-Rad) in a final volume of 10 μL/well.

The thermal profile of the PCR procedure following the SsoAdvancedsupermix protocol was: 1) Enzyme activation and initial DNA denaturation at 95°C for 30 seconds; 2) 5 seconds denaturation at 95°C, 20 seconds annealing and extension at 60°C repeated for 40 cycles (amplification data collected at the end of each cycle); 3) dissociation curve consisting of 5 seconds incubation at 65 – 95°C in 0.5°C increments. Melting curves were used to validate product specificity. The assay included no template (NTC), no template during RT (RT-), and no RT (NRT) controls to detect reagent contamination and presence of genomic DNA. Biological replicates from three independent experimental reproductions were separated and amplified in triplicate for the GOI and reference genes (one biological reproduction of each treatment sample with three technical replicates per plate). Calibration curves generated from a 10-fold dilution series of reverse transcribed cDNA (derived from T-47D cells cultured in 10% FBS) ranging from 50 ng to 5 pg cDNA/well were included in triplicate for every gene on every 384-well plate. An inter-run calibrator of this cDNA (1ng/well) was also included in triplicate for every gene on every 384-well plate. The slope of the linear equation generated from the calibration curve was applied to calculate the efficiency according to the equation E = (10[−1/slope]−1)×100. For data analysis, the quantification cycle (Cq) value was determined and specific gene expression normalized to exogenous controls using ΔΔCq method. Expression of RPS13 and PUM-1 genes were set as exogenous controls (Reference genes). The normalized ΔΔCq from treated samples was compared with the vehicle control (DMSO, C) to obtain values and used to calculate relative fold change.

### Cell Viability Assays

All growth studies were conducted in 12-well culture plates. To ensure active, non-confluent cell populations during treatment duration, 12-well culture plates were initially seeded with 3.0 × 10^4^ cells per well in 1 ml culture medium containing 10% FBS. Studies were limited to 7-day total duration to correlate with results of Western analyses. On the seventh day, the cells were trypsinized, and removed from individual wells of the culture plate. Cells were stained with propidium iodide and underwent imaging cytometry by the Nexcelom Cellometer Vision.

### Immunofluorescence and Confocal Microscopy

T-47D cells were plated on cover slips in 12-well plates (30,000 cells/well) and cultured for 48 hours in 10% FBS. The cell culture medium was then changed to 5% DCC-FBS and fresh medium was added at 2-day intervals. The cells were cultured in 5% DCC-FBS for a total of 4 days. On day 4, the ligands were suspended in 5% DCC-FBS media, and semi-confluent cells were treated for 24 hours.

Cellular localization of ERα by immunocytochemistry: the cells were fixed on cover slips for 10 minutes with 1% formalin in PBS, permeabilized with ice-cold acetone and methanol (50:50) and washed three times with PBS. Staining procedures were performed in a humidified chamber at 23°C. Cells were incubated in 10% goat serum (Sigma) to suppress nonspecific binding of IgG, followed by 3 hours incubation with 1:150 dilution anti-ERα (F-10) monoclonal antibody. After washing with PBS, cells were incubated for 3 hours with 1:200 dilution anti-mouse IgG conjugated with Cy3 (Jackson ImmunoResearch Laboratories, West Grove, PA). Cover slips were washed in PBS, and incubated for 2 minutes in 1 μg/ml 4′,6-diamidino-2-phenylindole (DAPI) dissolved in PBS. Cells were washed 3 times in PBS, mounted with Fluoromunt-G (Electron Microscopy Sciences, Hatfield, PA) and stored in the dark at 4°C.

Structural analysis of MTs by immunocytochemistry: Cells were fixed with PHEM buffer (68 mM PIPES, 25 mM HEPES, 15 mM EGTA, 3 mM MgCl_2_, pH 6.8) containing 3.7% formaldehyde, 0.05% glutaraldehyde, and 0.5% Triton X-100 for 10 minutes at room temperature. Coverslips were processed for direct-labeling immunofluorescence for 2 hours with Alexa Fluor 488 anti-tubulin-α antibody (Biolegend, San Diego, CA) diluted 4 μg/ml in 1% BSA with 0.05% Triton X-100 in PBS. DAPI nuclear counterstaining and mounting was performed as described above.

The distribution of three-dimensional fluorescent structures was analyzed using a Nikon Digital Eclipse C1 plus confocal microscope. NIS elements software (Nikon Instruments, Melville, NY) was used for noise reduction and three-dimensional reconstruction of the images.

### Statistical Analyses

The results are expressed as mean ± SEM. Statistical significance was determined by Kruskal-Wallis test followed by post-hoc analysis using Mann-Whitney U-test. p-values were adjusted for multiple testing corrections using the False Discovery Rate. Differences are considered significant at q < 0.05. Statistical analyses were carried out using SPSS for Windows version 11.5 (SPSS Inc., Chicago, IL).
